# Embryo ecology: Developmental synchrony and asynchrony in the embryonic development of wild annual fish populations

**DOI:** 10.1002/ece3.7402

**Published:** 2021-03-19

**Authors:** Matej Polačik, Milan Vrtílek, Martin Reichard, Jakub Žák, Radim Blažek, Jason Podrabsky

**Affiliations:** ^1^ Institute of Vertebrate Biology The Czech Academy of Sciences Brno Czech Republic; ^2^ Department of Zoology Charles University Prague Czech Republic; ^3^ Center for Life in Extreme Environments Portland State University Portland OR USA

**Keywords:** bet‐hedging, dormancy, egg bank, facultative, phenotypic plasticity

## Abstract

Embryo–environment interactions are of paramount importance during the development of all organisms, and impacts during this period can echo far into later stages of ontogeny. African annual fish of the genus *Nothobranchius* live in temporary pools and their eggs survive the dry season in the dry bottom substrate of the pools by entering a facultative developmental arrest termed diapause. Uniquely among animals, the embryos (encased in eggs) may enter diapause at three different developmental stages. Such a system allows for the potential to employ different regulation mechanisms for each diapause. We sampled multiple *Nothobranchius* embryo banks across the progressing season, species, and populations. We present important baseline field data and examine the role of environmental regulation in the embryonic development of this unique system. We describe the course of embryo development in the wild and find it to be very different from the typical development under laboratory conditions. Development across the embryo banks was synchronized within and across the sampled populations with all embryos entering diapause I during the rainy season and diapause II during the dry season. Asynchrony occurred at transient phases of the habitat, during the process of habitat desiccation, and at the end of the dry season. Our findings reveal the significance of environmental conditions in the serial character of the annual fish diapauses.

## Graphical abstract

Annual killifish of the African genus Nothobranchius are being widely used as laboratory model organisms. Nothing was known on their embryonic development under natural conditions. We bring first complete data on the natural course of embryonic development of the fish.

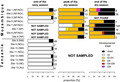

## 1 INTRODUCTION

Embryos often passively depend on their environment and can be extremely sensitive to environmental shifts. A subtle environmental insult during a key developmental window can have a major impact on embryo viability, and embryonic and postembryonic performance (e.g., Wilson, 1973). The embryonic phase is a critical component of ontogeny, and most vertebrates attempt to protect or shield their embryonic stages from environmental fluctuations through a variety of physiological and behavioral mechanisms such as internal incubation, careful oviposition choice, and parental care (e.g., Slater & Milinski, 1996).

In variable or highly seasonal environments, embryos often tolerate periods of unfavorable conditions in a state of dormancy. Embryonic dormancy has evolved in many plant, invertebrate, and vertebrate taxa (e.g., Cáceres, 1997; Childs et al., 2010; Hand et al., 2016). Diapause is a specific form of this dormancy which is under the control of an endogenous program. Its initiation precedes the onset of harsh conditions and may persist even after the harsh conditions recede (Hand, 1991; Strachan et al., 2015). Diapause may be initiated by specific environmental cues that precede unfavorable conditions (e.g., thermoperiod, food quality). Diapause entry and exit may also require cues which are not necessarily coincident with the return of favorable conditions in the habitat, such as photoperiod (Hand, 1991). Thus, while diapause is endogenously controlled, environmental factors are critical for triggering entry and exit from such dormancy and may regulate the responsiveness of embryos to environmental cues (Hand et al., 2016; Košťál, 2006).

Annual fishes of Africa and America represent a unique system to study the fundamental principles of the embryo–environment relationship. The fish lay drought‐resistant eggs in the bottom substrate of seasonally desiccating pools. They scatter their eggs and provide no parental care, yet their embryo incubation environment is extremely unstable and harsh. When the pool dries out, adult fish die but the eggs remain in the substrate and hatch in the next rainy season when the pools refill with water (e.g., Berois et al., 2015; Cellerino et al., 2016). The embryo incubation period imposes challenging conditions (anoxia, dehydration, temperature extremes) Podrabsky et al. (2016) and typically lasts several months (Reichard, 2015; Volcan et al., 2015). In fact, annual fish spend most of their lives as embryos.

Annual fish have evolved a system of three facultative dormant stages that occur during embryonic development (Levels & Denucé, 1988; Pinceel et al., 2015; Podrabsky et al., 2017, 2015; Wourms, 1972a, 1972c,1972a, 1972c). These are well‐defined and historically termed diapause I (DI), diapause II (DII), and diapause III (DIII). Diapause I may occur early in development before formation of the embryonic axis where the cells that will later form the embryo are more or less evenly dispersed across the yolk surface (Wourms, 1972b, 1972c,1972b, 1972c). Exit of DI is followed by reaggregation of the cells and formation of the embryonic axis. Diapause II may be entered about midway through development in the long somite embryo. DII embryos possess a full complement of somite pairs, a differentiated central nervous system, and a functional tubular heart with low heart rate frequency. Diapause III occurs in an essentially fully developed precocious embryo, awaiting a cue for hatching (Podrabsky et al., 2017; Wourms, 1972a). All three diapauses can be entered or skipped, and their duration is highly variable. The occurrence of three distinct embryonic diapause stages in a single taxon is unique to annual fishes (e.g., Podrabsky & Hand, 1999, 2015). Consequently, the system of three facultative diapauses (Levels & Denucé, 1988; Podrabsky et al., 2015) results in a multitude of potential developmental trajectories in annual fish termed the “multiplier effect”—a mechanism to maximize variation in embryonic developmental trajectory (Wourms, 1972c).

There is considerable ambiguity of the factors and mechanisms that regulate diapause dynamics in annual fishes. Laboratory research has identified several environmental factors that influence embryonic development. For example, diapause entry may be facilitated (and its duration prolonged) by low ambient temperature (DI, DII, and DIII; Markofsky & Matias, 1977), lack of oxygen (DI; Peters, 1963), and presence of adult fish (DI; Inglima et al., 1981). On the other hand, exit from a diapause may be triggered by an increase in ambient temperature (DI, DII, and DIII; Markofsky & Matias, 1977; Podrabsky et al., 2010) and presence of light (DII; Podrabsky & Hand, 1999; Romney et al., 2018) or simply by submersion in water following aerial incubation (DIII; Pinceel et al., 2015). At the same time, there is wide consensus that the generally unpredictable nature of a temporary pool ecosystem severely restricts reliability of environmental cues (Domínguez‐Castanedo et al., 2017; Furness et al., 2015; Pinceel et al., 2015; Polačik et al., 2017). If the entire embryo bank responds to the same cue by following the same developmental trajectory, synchronous development could result in a total recruitment failure (e.g., a synchronized hatching makes a population prone to be wiped out by a weak rainfall and subsequent premature desiccation of the habitat). One strategy to overcome a lack of environmental predictability is to have high levels of intrinsic variability within the embryo bank to spread risk and preclude reproductive failure. Existence of such an endogenously controlled developmental variation is strongly supported by data from the laboratory, where annual fish embryos incubated under the same conditions display great variability in their development (Furness, Lee, et al., 2015; Hu et al., 2020; Pinceel et al., 2015; Polačik et al., 2017; Wourms, 1972c). Such variability, likely of epigenetic origin (Romney & Podrabsky, 2017), is explained as an evolutionary bet‐hedging strategy (Furness, Lee, et al., 2015; Polačik et al., 2017; Simons, 2011), which ensures that at least part of the progeny develop optimally for a given set of actual conditions in the pool.

The mechanisms underlying diapause in annual fishes are complex, yet we have a very limited understanding of how diapause progresses in the wild, outside of the laboratory setting. Watters (2009) proposed a theory of season‐specific succession through the diapauses, according to which embryos (a) stay in DI in a pool yet inundated, (b) proceed to DII when the pool desiccates, and (c) exit DII and enter DIII at the end of the dry season, when the substrate is moistened by the first rains of the rainy season. The scheme is not built on direct sampling of embryo banks, as this was likely hampered by a lack of practical methodology, but instead grounded in a lifelong experience of the author as an enthusiastic annual fish hobbyist and trained geologist. These theoretical assumptions have been corroborated by the only sampling of a natural population of annual fish to date. Domínguez‐Castanedo et al. (2017) collected embryos of a single population of *Millerichthys robustus* (Miller and Hubbs), the only North American annual fish species, using a method of eye‐searching and hand‐picking the embryos from the substrate. They found embryos in DI in the inundated pool, in DII during the dry period and collected a mixture of DII and DIII embryos in moist substrate just prior to full inundation of the pool.

For the first time, we investigated natural embryonic development of African annual fishes of the genus *Nothobranchius*. One such species, the turquoise killifish *Nothobranchius furzeri* Jubb, is an important laboratory model, naturally occurring in southern Mozambique. It exhibits the typical processes of vertebrate aging compressed into a uniquely short period, making it very practical for experimental research (reviewed in Cellerino et al., 2016 and Hu & Brunet, 2018). Despite the knowledge that embryonic developmental trajectory of *N. furzeri* may have important consequences for posthatching lifespan (Polačik et al., 2014 but see Hu et al., 2020), embryonic development under natural conditions remains completely unexplored.

The main goal of our study was to test the hypothesis of environmentally driven progression through diapause stages as proposed by Watters (2009). Following this hypothesis, we predicted that *Nothobranchius* spp. (a) spend the inundation in DI; (b) progress to DII when the habitats desiccate; and (c) progress to DIII just before the onset of the next rainy season. We examine the linkage between habitat characteristics and within‐ and between‐population variability in embryo development during the progressing season. We also examined the applicability of laboratory‐based experimental findings to the natural incubation conditions of *Nothobranchius* spp. (Furness, Lee, et al., 2015; Pinceel et al., 2015; Podrabsky et al., 2007; Polačik et al., 2017; Wourms, 1972c).

## 2 METHODS

### 2.1 Study area and fish communities

We sampled 13 egg banks (pools) representing seven species of *Nothobranchius* in southern Mozambique and coastal Tanzania. All the sampled populations represented typical *Nothobranchius* habitats in terms of abiotic and biotic parameters. In Mozambique, regular occurrence of the fish in the sampled pools has been documented since 2008 (e.g., Polačik et al., 2014; Reichard et al., 2009; Vrtílek et al., 2018). The consistency of fish occurrence in the Tanzanian study area and sites was confirmed by our previous trip (Bartáková et al., 2020) and repeated trips by several hobby collectors (e.g., Nagy & Kis, 2010; Shidlovsky, 2010).

In Mozambique, we sampled eight pools within the range of *N. furzeri* (Reichard et al., 2009) inhabited by a combination of *N. furzeri*, *Nothobranchius orthonotus* (Peters), and *Nothobranchius pienaari* (Shidlovskyi, Watters and Wildekamp). The sampling campaign covered the entire embryonic phase of the fishes’ lifecycle—the end of the rainy season (March 2018), the peak of the dry season (July 2018), and the end of the dry season (December 2018; Table 1). The sampling in Tanzania took place at the end of the long rainy season (June 2019). We sampled five inundated pools with communities combining *Nothobranchius eggersi* Seegers, *Nothobranchius janpapi* Wildekamp, *Nothobranchius melanospilus* (Pfeffer), and *Nothobranchius ocellatus* (Seegers).

**TABLE 1:** Data on sites sampled in Mozambique

Site no.	GPS	Fish community	Species proportion (%)	Area (m^2^)	Depth (cm)	River basin	*W*_ers_ (kg)	*W*_pds_ (kg)	*W*_eds_ (kg)	Embryo presence/absence
1	24°9′35.16″S	*N. furzeri*	100	25	20	Limpopo North	30 (5)	40 (4)	51 (4)	+
	32°48′3.54″E									
2	24°9′35.16″S	*N. furzeri*	100	12	50	Limpopo North	30 (3)	40 (2)	61 (4)	+
	32°48′3.54″E									
3	24°9′35.16″S	*N. furzeri*	100	4	10	Limpopo North	30 (1)	20 (1)	18 (3)	+
	32°48′3.54″E									
4	24°18′44.16″S	*N. furzeri*	80	100	30	Limpopo North	20 (4)	—	—	−
	33°2′11.94″E	*N. orthonotus*	20							
5	23°28′24.36″S	*N. furzeri*	90	300	40	Limpopo North	30 (5)	8 (1)	109 (5)	+
	32°34′1.08″E	*N. orthonotus*	10							
6	22°44′22.86″S	*N. furzeri*	100	200	20	Chefu	40 (6)	80 (4)	—	—
	32°6′23.16″E									
7	24°19′36.53″S	*N. furzeri*	45	1,100	60	Limpopo South	—	192 (11)	111 (10)	+
	32°42′25.13″E	*N. orthonotus*	45							
		*N. pienaari*	10							
8	24°7′38.46″S	*N. furzeri*	60	1,200	70	Limpopo North	—	20	101 (4)	+
	32°46′54.90″E	*N. orthonotus*	30							
		*N. pienaari*	10							

Note

*W*_ers_; *W*_pds_; *W*_eds_ = total weight of soil sample processed at the end of the rainy season, peak of the dry season, and end of the dry season, respectively. Species proportion = species proportions in the given *Nothobranchius* community, sampled simultaneously with the embryos. Parentheses = number of separate samples from each site and season.

The bottom substrate always consisted of a dark clay soil (likely vertisol soils according to Watters, 2009) mixed with a minority proportion of very fine quartz sand (estimated to be 5%–15%) and organic debris (estimated to be 1%–10%). Inundated pool size ranged from 4 to 1,200 m^2^ (Tables 1 and 2) at the end of the rainy season sampling. We targeted pools with high densities of adult fish during the inundation. Adult *Nothobranchius* spp. community was sampled using a dip net and a seine net (see Vrtílek et al., 2018 for details). Sites 7 and 8 were not sampled during inundation since only juvenile fish not old enough to produce embryos were present during that campaign. Sampling of parental fish communities during the wet phase also served as a reference for expected species diversity of the embryo bank (Tables 1 and 2).

**TABLE 2:** Data on sites sampled in Tanzania

Site no.	GPS	Community	Species proportion (%)	Area (m^2^)	Depth (cm)	River basin	W_ers_ (kg)	W_pds_ (kg)	W_eds_ (kg)	Embryo presence/absence
9	8°4′29.16″S	*N. annectens*	8	150	30	Rufiji	20 (1)	—	—	+
	39°0′8.40″E	*N. janpapi*	23							
		*N. melanospilus*	63							
		*N. ocellatus*	6							
10	7°53′11.70″S	*N. janpapi*	4	80	30	Rufiji	20 (1)	—	—	+
	38°41′47.34″E	*N. melanospilus*	96							
11	8°9′27.24″S	*N. janpapi*	12	150	70	Rufiji	20 (1)	—	—	+
	39°10′54.60″E	*N. melanospilus*	88							
12	6°28′2.76″S	*N. eggersi*	4	60	40	Ruvu	20 (1)	—	—	+
	38°47′55.26″E	*N. janpapi*	13							
		*N. melanospilus*	80							
		*N. ocellatus*	3							
13	6°28′55.92″S	*N. eggersi*	62	54	30	Ruvu	10 (1)	—	—	+
	38°54′51.51″E	*N. janpapi*	17							
		*N. melanospilus*	21							

Note

Water area and depth reflect situation during the sampling at the end of the rainy season. *W*_ers_; *W*_pds_; *W*_eds_ = total weight of soil sample processed at the end of the rainy season, peak of the dry season, and end of the dry season, respectively. Species proportion = species proportions of adult fish in the given *Nothobranchius* community, sampled simultaneously with the embryos. Parentheses = number of separate samples from each site and season.

Ambient temperature has been recognized as an important environmental factor influencing development and survival of annual fish embryos (e.g., Markofsky & Matias, 1977; Romney et al., 2018). We used temperature data loggers (HOBO Pendant^®^ Temperature Data Logger UA‐002‐08, Onset) to continually monitor substrate temperature in Mozambique in four *Nothobranchius* pools throughout the entire period of embryonic development. A set of three data loggers per site was buried in four pools (12 data loggers in total). The data loggers were placed into the wet mud during the first sampling campaign (March, inundated pools) to allow for natural encasement in the substrate. The exposure depth was 5, 10, and 20 cm in the substrate. The loggers were read at the peak of the dry season (to ensure at least partial data availability in case of a logger loss before the final reading) and then re‐deployed to obtain the final dataset at the end of the dry season.

Annual fish embryos are extremely tolerant to laboratory‐induced anoxia (e.g., Podrabsky et al., 2007), but data on oxygen availability from their natural habitat are lacking. We deployed data loggers (HOBO Dissolved Oxygen Data Logger U26‐001, Onset) to measure oxygen concentration within 5 cm depth in the substrate of three inundated *Nothobranchius* spp. pools in Mozambique. We opted to use a long‐term exposure data logger (instead of an instantly measuring oxygen meter) to obtain more relevant data, unaffected by the initial substrate disturbance when burying the probe. The measuring interval was 0.5 hr, and the duration of exposure ranged from 15 to 27 hr. Additionally, we measured oxygen availability just above the substrate (50 cm depth) at site 7 to assess the contrast between the substrate and the water column.

Survival of aerial incubation in annual fish embryos is highly influenced by relative humidity under laboratory conditions (Podrabsky et al., 2001). We measured soil moisture (relative humidity in %) in the visually dry substrate at the peak and the end of the dry season using a soil moisture probe (Extech MO750, Extech). Soil moisture was measured in the central region of a desiccated pool. The number of measured spots varied from one to 32 per pool (higher number of measurements occurred during the process of method optimization) and the depth of measurements from 5 to 20 cm.

### 2.2 Embryo sampling

*Nothobranchius* spp. embryos were extracted from the bottom substrate by a method of soil liquefaction. We added water to the substrate and subsequently sieved the liquefied soil through a fine mesh sieve. We then collected the embryos from the sieve and determined their developmental stage in the field.

Bottom substrate was sampled using a shovel (inundated pool) or a pickax (dry pool). The depth of the samples varied from 5 to 15 cm. The number of soil samples (1–11) and sample weight (3–30 kg) per pool was variable as we always aimed to obtain a numerically representative sample from sites with varying embryo density. More intensive sampling was performed in particular pools during method optimization, vertical distribution sampling (see below) and to maintain sample continuity across successive sampling campaigns (Table 1). When sampling a habitat in a dry phase, we tried to increase the chance of obtaining sufficient numbers of embryos by targeting parts of the bottom which showed clear signs of preceding inundation (such as the presence of deep cracks and absence of terrestrial vegetation).

Each soil sample was mixed with water until it became liquefied. The volume of water added equaled the volume of the substrate in wet soil samples (end of the rainy season), whereas in the dry substrate, the volume of the water added was about 150% of the sample volume. The mixture was briefly stirred and about 3 L of the suspension poured through a sieve (20 cm in diameter, 0.8 mm mesh size) at a time. The sieve was then partially submerged in a container of clean water and the contents stirred by hand to wash away excess soil. Embryos retained in the sieve were identified by eye using a light source placed over the sieve to increase contrast.

The collected embryos were cleaned of debris to improve visibility of embryonic structures. We determined their developmental stage using portable microscopes (Specwell M0616‐E 6x16, LS & S; and BoliOptics 40‐400X) within up to three hours after soil liquefaction. We assigned each embryo into one of six categories, following Wourms (1972a) and Podrabsky et al. (2017): (a) DI embryo (embryo lacking visible embryonic axis, potentially including pre‐DI and early post‐DI embryos, stage 2‐26); (b) post‐DI–pre‐DII embryo (stage 27‐32), (c) DII embryo (stage 33); (d) post‐DII–pre‐DIII embryo (stage 34‐40); (e) DIII embryo (full size, gold colored iris, stage 41‐43); and (f) dead embryo (embryo with opaque or collapsed interior).

**FIGURE 1:**
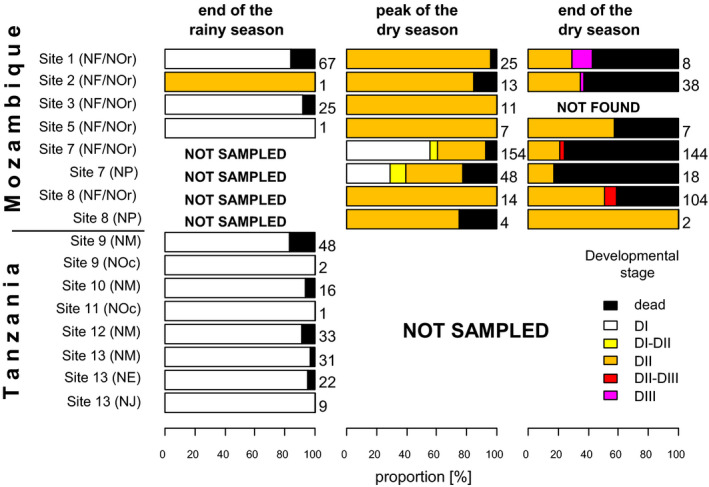
*Nothobranchius* spp. embryo proportions according to the developmental stage and embryo mortality at the end of the rainy season (Mozambique, Tanzania), peak of the dry season, and end of the dry season (Mozambique). Number of embryos per site is given next to the each of the barplots. NF = *N. furzeri*; NOr = *N. orthonotus*; NP = *N. pienaarii*; NM = *N. melanospilus*; NOc = *N. ocellatus*; NE = *N. eggersi*; NJ = *N. janpapi*

Pilot sampling revealed that *Nothobranchius* spp. embryos were distributed in the substrate across a range of depths. We collected specific, vertically layered substrate samples from high embryo density sites 7 and 8 (Figure 1; to increase the chance of successful embryo collection) to determine the vertical distribution of embryos. Site 7 was sampled at the peak of the dry season. The substrate was collected within a 30 × 30 cm square in three descending layers (<5, 5–10, and 10–15 cm deep). Three replicated samples (three squares) were obtained and separately processed. At the end of the dry season, we repeated the vertical sampling at sites 7 and 8 using the same approach. A single vertical sample (30 × 30 cm square) was processed in each of these two sites.

We determined species affiliation of the embryos according to species‐specific egg size differences (Eckerström‐Liedholm et al., 2017; Vrtílek & Reichard, 2016; Watters et al., 2020) and information on the occurrence of *Nothobranchius* species in the respective pools during the inundation period. We assigned each embryo into a putative species during the developmental stage determination and preserved the embryos in 4% formaldehyde for a size measurement in the laboratory (Vrtílek & Reichard, 2016). We performed a confirmatory analysis based on size frequency distribution of each species. While this was possible for all four Tanzanian species (Watters et al., 2020), out of the three Mozambican species, we only could clearly separate *N. pienaari* from *N. furzeri* and *N. orthonotus*, because the latter two show an overlap in egg size distribution (Eckerström‐Liedholm et al., 2017; Vrtílek & Reichard, 2016). Species affiliations were additionally verified by hatching a subsample of eggs and raising the fish until the species could be reliably determined.

### 2.3 Data analysis

We tested the effect of depth on substrate moisture between the peak and the end of the dry season. The analysis was performed in two steps using a linear mixed effect model (LMM) (function “lmer” from package “lme4” v 1.1.21.; Bates et al., 2015). Soil moisture data approached normal distribution after square‐root transformation. Step 1 was done on the dataset with all sites, and Step 2 was done on a dataset excluding site 7, as it was still damp during the peak of the dry season. In both models, *soil moisture* was used as a response variable. *Season* and *depth* were used as predictors in interaction, and *site* was introduced as a random factor with random slope. The introduction of *depth* as a random slope did not improve model fit (ΔAIC < 2) and was omitted. Assumption on normality of model residuals was checked using diagnostic plots. Statistical analysis was done in R environment v 3.6.1. (R Core Team, 2019).

## 3 RESULTS

### 3.1 Species composition in the egg banks

A total of 691 and 162 *Nothobranchius* spp. embryos were examined during the survey in Mozambique and Tanzania, respectively. Embryos were recovered from 11 out of 13 sampled pools that were populated with annual fish during that particular rainy season (Tables 1 and 2; Figure 1). In Mozambique, the egg banks were dominated by embryos of *N. furzeri/N. orthonotus* (89.6%) with a low proportion of *N. pienaari* (10.4%; Figure 1). The composition of the egg banks reflected relative species abundance in the parental generation (Table 1). In Tanzania, embryos of *N. melanospilus* were the most abundant (79%), followed by *N. eggersi* (13.6%), *N. janpapi* (5.6%), and *N. ocellatus* (1.8%; Figure 1). The egg bank species composition corresponded well with the species proportions in the parental generation except for *N. janpapi*, embryos of which were rarely found (Table 2).

### 3.2 Seasonal embryo development and mortality

All the sampled pools were still inundated at the end of the rainy season, and all live embryos but one (found outside the substrate, attached to a plant stem) were in DI (Mozambique: 98.8% DI, 1.2% DII; Tanzania: 100% in DI; *n* = 256; Figure 1).

**FIGURE 2:**
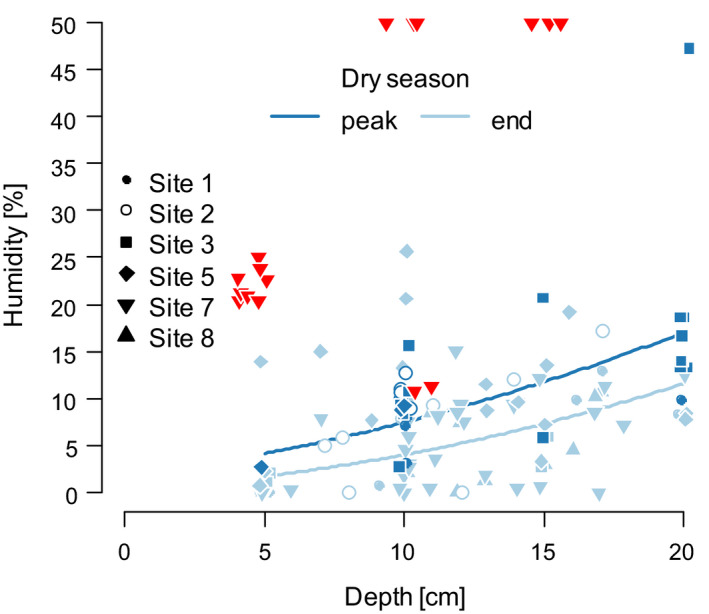
Bottom substrate humidity according to the season and depth in six *Nothobranchius* spp. pools (Mozambique). Site 7 was still damp at the peak of the dry season (red reversed triangles), and the humidity values were not included in the regression

At the peak of the dry season (Mozambique only), five of the study sites were dry, but bottom substrate at site 7 was still saturated with water (Figure 2). Egg banks at the five dry sites consisted invariably of DII‐stage embryos, while, at site 7, we recorded developmental variation with 59.4% embryos in DI, 5.6% in between DI and DII, and 35% in DII. There was no qualitative difference between the developmental progression of *N*. *furzeri/N. orthonotus* and *N. pienaari* (Figure 1).

Near the end of the dry season, the sampled sites were on average drier than at the peak of the dry season (Figure 2). The egg banks showed variability in embryo developmental stage with 88.4% embryos in DII, 9.9% between DII and DIII, and 1.7% in DIII. Again, we did not record any qualitative difference between the development of *N*. *furzeri/N. orthonotus* and *N. pienaari* (Figure 1).

The proportion of dead embryos was generally low at the end of the rainy season (Mozambique: 14%; Tanzania: 8.6%) and at the peak of the dry season (9.4%) but considerably increased at the end of the dry season (62%). The trend was obvious across all sites and species (Figure 1).

### 3.3 Vertical embryo distribution

**FIGURE 3:**
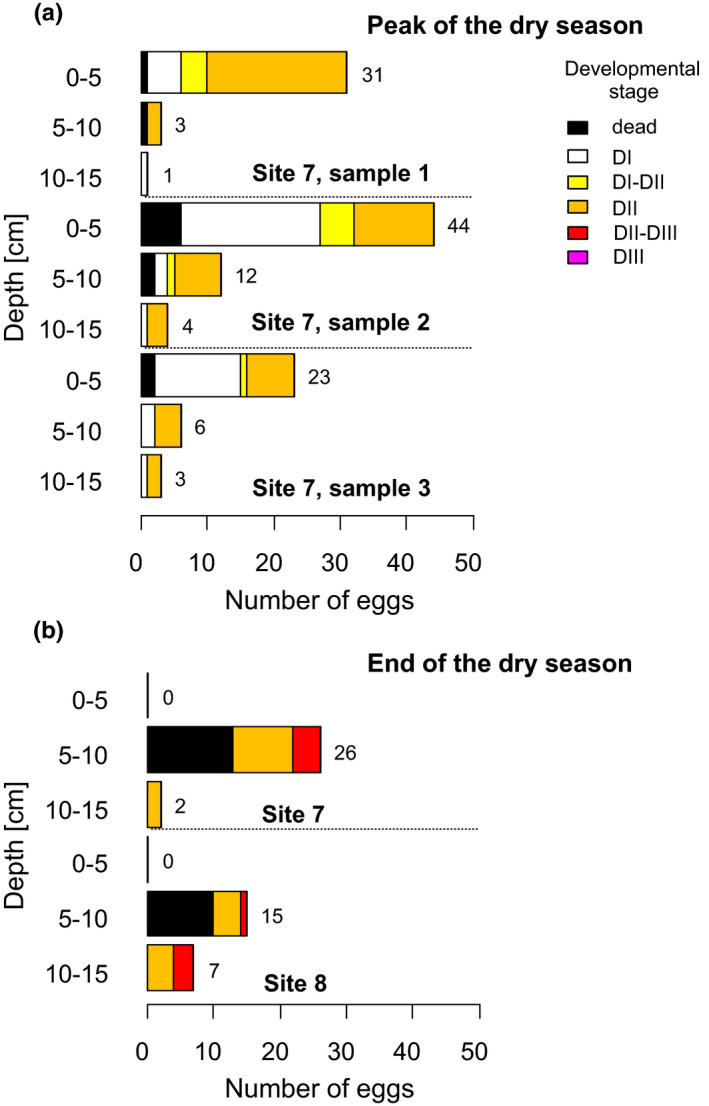
Vertical distribution of *Nothobranchius* spp. embryos in the substrate at the peak (a) and end (b) of the dry season (Mozambique). Number of embryos per site is given next to the each of the bar plots

At the peak of the dry season, live *Nothobranchius* spp. embryos were found throughout the entire examined soil core (0–15 cm) of site 7 but embryo abundance appeared to decrease with the depth (Figure 3a).

**FIGURE 4:**
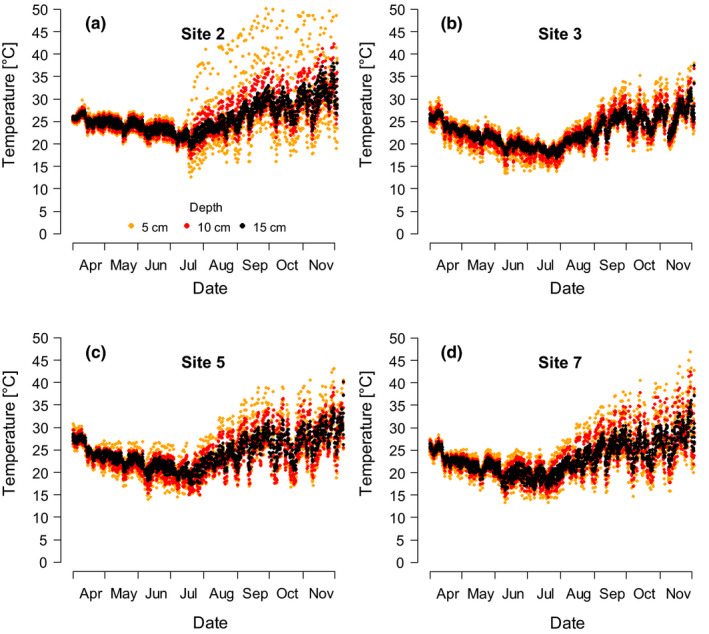
Seasonal course of temperature change in the bottom substrate of four *Nothobranchius* spp. pools (a, b, c, d) at 5, 10, and 15 cm (Mozambique). Please note that measured values at site 2 (a) showed abnormally high amplitude caused by inconsistent re‐exposure of the data logger after its reading on July 15th and should be taken with caution

At the end of the dry season, live embryos were only found in deeper substrate layers (>5 cm depth; Figure 3b). The uppermost layer (down to the 5 cm depth) appeared eroded in both sampled sites (sites 7 and 8). The formerly hard, top surface layer with deep cracks turned into a uniform dusty coating, with the cracks disappearing. Soil moisture measurements for this layer thus invariably indicated zero moisture levels and daily soil temperature was peaking at over 40°C (Figure 4).

### 3.4 Abiotic conditions in the egg bank habitats

Substrate temperature ranged from a minimum of 13°C at the peak of the dry season to a maximum of 47°C at the end of the dry season (Figure 4d). Temperature stability slightly increased with depth and the seasonal course of temperature change followed the same pattern in all sites (Figure 4). Although even more extreme values were recorded (Figure 4a), these are likely to be a consequence of an inconsistent re‐exposure of the data logger set (abrupt change of the magnitude after July 15th).

**FIGURE 5:**
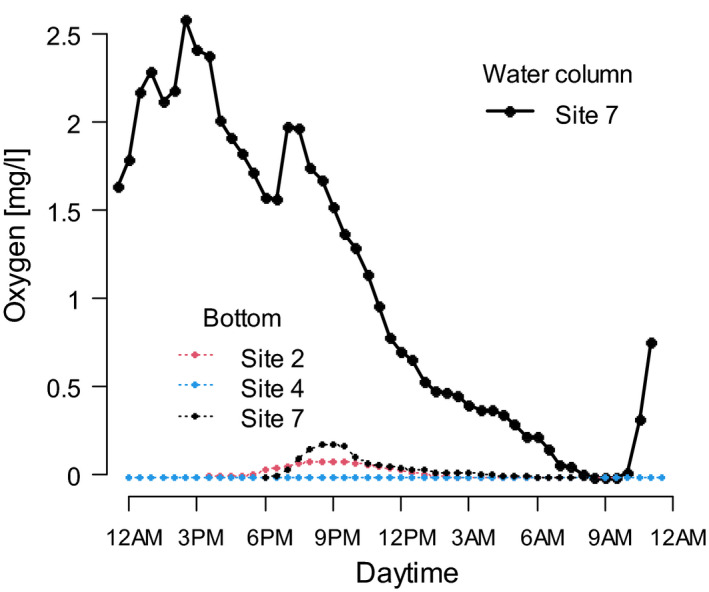
Oxygen concentration inside the bottom substrate (site 2; 4 and 7) and water column (site 7) of *Nothobranchius* spp. pools. Please note that the nonzero oxygen concentration values measured inside the bottom substrate are below the accuracy of the measuring device (±0.2 mg/L)

We recorded an absence of oxygen in the bottom substrate during the end of the rainy season. The values recorded from three pools were consistently below the measurement accuracy of the device (0.2 mg/L). In contrast, dissolved oxygen was available in the water column just above the substrate and its concentration changed across the course of a day (Figure 5).

Substrate moisture during the peak of the dry season was higher compared to the end of the dry season even when the outlier (site 7, substrate still saturated with water; Figure 2) was excluded from the analysis (effect of season, χ^2^ = 5.9, *df* = 1, *p* = .015, *N* = 75; after removing depth: season interaction, χ^2^ = 0.1, *df* = 1, *p* = .769). Soil moisture significantly increased with depth (χ^2^ = 25.5, *df* = 1, *p* < .001).

## 4 DISCUSSION

For the first time, we sampled egg banks of wild populations of *Nothobranchius* to obtain baseline data on the natural course of their embryonic development. During inundation and peaking dry phase, the embryo bank was characterized by strict developmental synchrony within a population. Inundated Mozambican and Tanzanian embryo banks of different *Nothobranchius* species showed the same developmental profile despite the geographic distance. Variability was only observed during the process of pool desiccation and at the end of the dry season. Substantial embryo mortality occurred at the end of the dry season, suggesting that the embryonic period is important for population recruitment success in the next rainy season. Overall, our data suggested an important role for environmental control over a major part of the embryonic cycle and the possibility of intrinsic developmental bet‐hedging potentially acting late in the dry season.

### 4.1 Signs of convergent evolution

Our findings are in accordance with the theoretical developmental scheme for *Nothobranchius* embryos proposed by Watters (2009) as well as the only field data so far available for an annual fish, *Millerichthys robustus* from Mexico (Domínguez‐Castanedo et al., 2017). Similar to *M. robustus*, our *Nothobranchius* embryo banks were composed of DI embryos during soil inundation, DII embryos during the peak of the dry season (except the water‐saturated site 7), and a mixture of DII with a minor proportion of post‐DII stages close to the end of the dry season. The similarity in the course of development between phylogenetically distant African and American species that have evolved annual lifestyle independently (Furness et al., 2015; Helmstetter et al., 2016) raises the question on the universality of the developmental sequence for annual fish in general. Efforts to collect data for temperate South American annual fish species (where, in contrast to our study region in Africa, the warm summer period rather than cold winter period coincides with desiccation) are ongoing.

### 4.2 The degree of developmental synchrony

The prominent developmental synchrony observed in multiple independent embryo banks during the inundation and at the peak of the dry season (Figure 3) suggests that there was a unifying environmental influence. Similar developmental synchronization is also known to occur in the laboratory when extreme conditions such as very low or high temperatures are applied (Dodzian et al., 2018; Furness, Lee, et al., 2015; Levels & Denucé, 1988; Markofsky & Matias, 1977), suggesting that the environment has the potential to govern the embryonic development of *Nothobranchius* spp. The main habitat phases in the wild appear to be reliable enough to favor canalization of developmental trajectories (Furness, Lee, et al., 2015).

The variability observed during the transitional habitat phases (the moist site 7 at the peak of the dry season and the end of the dry season as a whole) supports the hypothesis of Furness, Lee, et al. (2015) that an intrinsic bet‐hedging strategy can be employed under uncertain conditions when the transition may proceed in its expected direction but also revert back to the former state (re‐inundation or re‐drying). A diapause may be exited or maintained. Under these transitioning conditions, bet‐hedging in the form of developmental variability ensures that at least part of the progeny is matched with the forthcoming environment (Furness, Lee, et al., 2015; Simons, 2011). Alternatively, the variability seemingly manifested as intrinsic bet‐hedging could be a consequence of an incremental change in incubation microconditions. The microhabitat of each individual embryo is likely to impose continual but gradual changes (e.g., progressive drying from surface to deeper substrate layers during desiccation), resulting in asynchronous developmental progress in the embryo bank as a whole. However, the character of our data does not allow for full discrimination between the two alternative hypotheses and mechanistic studies that replicate natural conditions in the laboratory are needed to disentangle the question.

### 4.3 Regulation of the developmental process

In theory, the entry and exit of each of the three diapauses allows for differential regulation to achieve maximum survival during different phases of the seasonal cycle. Provided that an environmental signal is clear and unambiguous, environmental control is advantageous as it enables a direct response to actual conditions. On the other hand, an environmental uncertainty favors intrinsic bet‐hedging.

We found *Nothobranchius* spp. embryos to invariably reside in DI during pool inundation. Diapause I is a developmental stage specifically adapted to hypoxia (Podrabsky et al., 2007) in the substrate of water‐filled pools (Figure 5), which reliably signals lasting inundation. Lack of oxygen has already been suggested as the factor triggering DI entry (Inglima et al., 1981; Levels et al., 1986; Peters, 1963), but common methods of laboratory incubation prevent occurrence of anoxia (e.g., Dodzian et al., 2018; Polačik et al., 2016). This might explain why DI is very rare in captive *Nothobranchius* spp. (Levels & Denucé, 1988).

At the peak of the dry season, embryos were halted in DII. After the pools dry out, oxygen availability is re‐established (Watters, 2009), which is a clear cue about the loss of water from the habitat (and/or a lifted constraint of the previous lack of oxygen). Oxygen availability has been suggested to be the factor triggering DI exit (Peters, 1963). The embryos then proceeded to DII, a stage specifically adapted to dry conditions (Podrabsky et al., 2001). We hypothesize that the factor that invariably maintained all the examined embryos in DII and prevented their progression to DIII (Figure 3) might be the low ambient temperature. Low temperature is known to be a reliable trigger of DII entry in *Nothobranchius* spp. under laboratory conditions (e.g., Furness, Lee, et al., 2015; Markofsky & Matias, 1977; Polačik et al., 2016). The peak of the dry season represents the coolest part of the year in southern Mozambique, with temperatures regularly dropping below 15°C (Figure 4).

A mixture of DII, post‐DII, and DIII stages was collected at the end of the dry season. In contrast to the reliable cues of inundation and desiccation (see above), the signals that might inform the embryos of the approaching onset of the rainy season appear to be more ambiguous. For example, a weak initial rainfall might present a false hatching cue for embryos already residing in DIII. Developmental regulation being superseded by intrinsic bet‐hedging therefore makes sense at this part of the cycle.

### 4.4 Vertical distribution and mortality

Although *Nothobranchius* embryos were more abundant in the upper substrate layers, they were also recovered from as deep as 15 cm. In contrast to South American annual fishes (e.g., García et al., 2008; Papa et al., 2015), all involved *Nothobranchius* fishes are not substrate divers and position their eggs very close to the surface of the spawning substrate (e.g., Cellerino et al., 2016; Polačik et al., 2016). Hence, the occurrence of embryos at greater depths can be best explained by disturbance of the substrate caused by large mammals (primarily cattle in our study area, but presumably wild animals in natural areas), frequently visiting pools (M. Polačik pers. obs.). Occurrence of live embryos throughout the top 15 cm of substrate together with no survival in the uppermost layer at the end of the dry season suggests that this postspawning positional drift may affect embryo survival. The high mortality in the upper layer was likely caused by a combination of high temperatures (Figure 4; Matias & Markofsky, 1978; Podrabsky et al., 2015) and lack of humidity (Podrabsky et al., 2001). Notably, rainy season rainfall is not the sole determinant of the substrate humidity, as some smaller off‐seasonal precipitation is typical for the Southern Mozambique (Westerink, 1996, M. Polačik and R. Blažek pers. obs.) and may be important for embryo survival. Nevertheless, the substantial embryo mortality that we observed suggests that recruitment success in annual fishes may be strongly determined during embryonic development.

### 4.5 Potential limitations of the study

Our data are correlative and do not reveal direct causal links between the underlying ecological factors and embryonic development. We think that despite the uncontrolled character of our large‐scale field study, the main findings are robust and generalizable. They were replicated across populations, species, and even distant geographic regions (Southern Mozambique and the coastal part of Tanzania). We revealed clear developmental patterns associated with changes in environmental conditions, which are supported by mechanistic studies from the laboratory (e.g., Peters, 1963).

Time management trade‐offs and challenging conditions during the field survey (only a small, portable microscope available) required a simplified approach to embryo stage determination. We acknowledge that the absence of an embryonic axis is not the ultimate indication that an embryo was residing in DI (see Embryo sampling in Methods). Thus, our DI embryo category could potentially include pre‐DI and very early post‐DI stages (see Podrabsky et al., 2017 for details). However, we believe that the proportion of such erroneous determinations was negligible (if any) because *Nothobranchius* spp. embryos that do not enter the DI necessarily show a visible embryonic axis within 5–8 days after the fertilization (Dolfi et al., 2019; Wourms, 1972b, M. Polačik unpubl.data) under the temperature conditions in the substrate at that season (Figure 4). We sampled the nine egg banks at both geographic locations (Figure 1) at the end of the rainy season, presumably after months of continuous spawning of the fish (Vrtílek et al., 2018). It is therefore highly unlikely that all the collected eggs from the inundated pools were freshly laid (i.e., before the DI stage) while all the rest already decayed. Statistical probability of collecting virtually all our sample embryos (227 live individuals) in the very narrow time window between the spawning and before the axis formation is extremely low. Considering these indirect but independent lines of evidence, the large majority of embryos from category “DI” can be presumed to be in that developmental stage.

## 5 CONCLUSION

Our data on the natural embryonic development of *Nothobranchius* spp. offer a baseline for interpretation and design of research utilizing embryonic stages of this emerging laboratory model. To date, outcomes of laboratory‐based observations on *Nothobranchius* embryos have been frequently used to explain embryonic adaptations of annual fishes in their natural ecosystem (e.g., Furness, Lee, et al., 2015; Pinceel et al., 2015; Polačik et al., 2017). Polačik et al. (2014) found that the mode of embryonic development influences posthatching performance. For example, fish hatched from embryos with short development grew faster, matured earlier, and died sooner than the phenotypes originating from the embryos with long development. It appears important in this regard that according to our current data, the typical developmental patterns in captivity differ from development in situ. First and foremost, in the wild, the embryos tend to develop synchronously over a considerable part of the seasonal cycle. In captivity, the typical course of development is asynchronous (e.g., Furness, Lee, et al., 2015; Pinceel et al., 2015; Podrabsky et al., 2010; Polačik et al., 2016, 2017; Wourms, 1972a, 1972b, 1972c,1972a, 1972b, 1972c,1972a, 1972b, 1972c). This suggests a significant underappreciation of the importance and perhaps the reliability of environmental cues in the wild, despite the highly variable and seemingly unpredictable nature of annual fish habitat (Polačik et al., 2017). Ecological factors acting under natural conditions (e.g., clay‐like substrate, anoxia, seasonal and diurnal temperature, and moisture fluctuations) represent a developmental environment markedly distinct from dry and liquid‐media incubation techniques commonly employed in the laboratory (e.g., Dodzian et al., 2018; Polačik et al., 2016). It is possible that the array and intensity of factors acting in the wild to a large extent override variability in endogenous programming. In contrast, it seems the environmental influence of artificial incubation conditions is typically insufficient to overcome the genetic and epigenetic underpinning and the intrinsic developmental code prevails.

## CONFLICT OF INTEREST

The authors declare no conflict of interests.

## AUTHOR CONTRIBUTION

**Matej Polačik:** Conceptualization (equal); Data curation (equal); Formal analysis (equal); Funding acquisition (equal); Investigation (equal); Methodology (equal); Project administration (equal); Supervision (equal); Visualization (equal); Writing‐original draft (equal); Writing‐review & editing (equal). **Milan Vrtilek:** Conceptualization (equal); Data curation (equal); Formal analysis (equal); Investigation (equal); Methodology (equal); Visualization (equal); Writing‐original draft (equal); Writing‐review & editing (equal). **Martin Reichard:** Investigation (equal); Methodology (equal); Supervision (equal); Writing‐original draft (equal). **Jakub Zak:** Conceptualization (equal); Data curation (equal); Formal analysis (equal); Investigation (equal); Methodology (equal); Visualization (equal); Writing‐original draft (equal). **Radim Blazek:** Conceptualization (equal); Investigation (equal); Methodology (equal); Writing‐original draft (equal); Writing‐review & editing (equal). **Jason Earl Podrabsky:** Conceptualization (equal); Investigation (equal); Methodology (equal); Resources (equal); Validation (equal); Visualization (equal); Writing‐original draft (equal); Writing‐review & editing (equal).

## Data Availability

Original data from this study are available on Dryad repository: https://doi.org/10.5061/dryad.cc2fqz65k.
